# Scientometric Analysis of Artificial Intelligence (AI) for Geohazard Research

**DOI:** 10.3390/s22207814

**Published:** 2022-10-14

**Authors:** Sheng Jiang, Junwei Ma, Zhiyang Liu, Haixiang Guo

**Affiliations:** 1 Badong National Observation and Research Station of Geohazards (BNORSG), China University of Geosciences, Wuhan 430074, China; 2 Three Gorges Research Center for Geohazards of the Ministry of Education, China University of Geosciences, Wuhan 430074, China; 3 School of Economics and Management, China University of Geosciences, Wuhan 430074, China

**Keywords:** geohazard, artificial intelligence (AI), scientometric, visualization, research cluster

## Abstract

Geohazard prevention and mitigation are highly complex and remain challenges for researchers and practitioners. Artificial intelligence (AI) has become an effective tool for addressing these challenges. Therefore, for decades, an increasing number of researchers have begun to conduct AI research in the field of geohazards leading to rapid growth in the number of related papers. This has made it difficult for researchers and practitioners to grasp information on cutting-edge developments in the field, thus necessitating a comprehensive review and analysis of the current state of development in the field. In this study, a comprehensive scientometric analysis appraising the state-of-the-art research for geohazard was performed based on 9226 scientometric records from the Web of Science core collection database. Multiple types of scientometric techniques, including coauthor analysis, co-citation analysis, and cluster analysis were employed to identify the most productive researchers, institutions, and hot research topics. The results show that research related to the application of AI in the field of geohazards experienced a period of rapid growth after 2000, with major developments in the field occurring in China, the United States, and Italy. The hot research topics in this field are ground motion, deep learning (DL), and landslides. The commonly used AI algorithms include DL, support vector machine (SVM), and decision tree (DT). The obtained visualization on research networks offers valuable insights and an in-depth understanding of the key researchers, institutions, fundamental articles, and salient topics through animated maps. We believe that this scientometric review offers useful reference points for early-stage researchers and provides valuable in-depth information to experienced researchers and practitioners in the field of geohazard research. This scientometric analysis and visualization are promising for reflecting the global picture of AI-based geohazard research comprehensively and possess potential for the visualization of the emerging trends in other research fields.

## 1. Introduction

**Figure 1:**
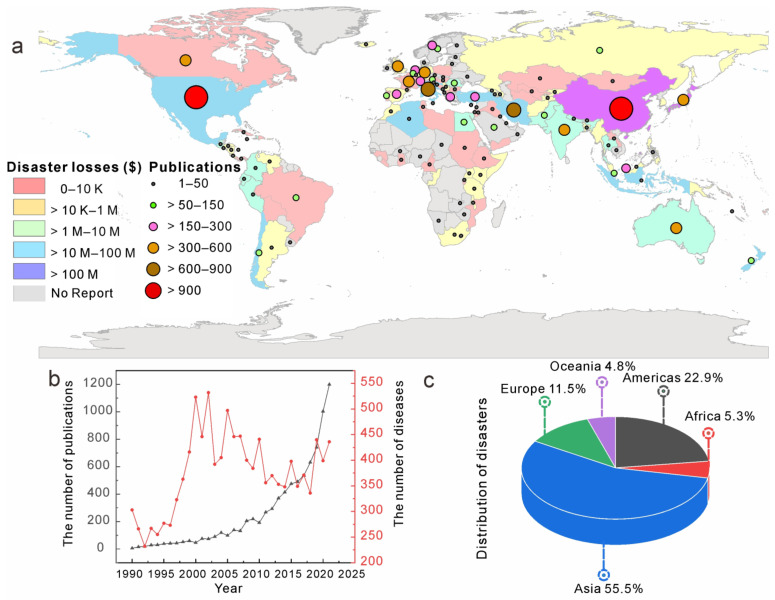
(**a**) The distribution of geohazard loss (Source: https://public.emdat.be/data, accessed on 7 July 2022) and the number of papers. Different colors indicate different degrees of geohazard loss and the size and color of the circles indicate the number of papers published in that country. (**b**) The change in the number of geohazards and the number of publications over time. (**c**) The regional distribution of the number of geohazards.

According to the Occupational Safety and Health Administration (OSHA, https://www.ccohs.ca/oshanswers/hsprograms/hazard_risk.html, accessed on 3 October 2022), a hazard is any source of potential damage, harm, or adverse health effects on something or someone under certain conditions at work. Geohazards refer to events caused by geological conditions or processes that pose a threat to human life, property, or the natural environment [1]. According to the Emergency Events Database (EM-DAT, https://public.emdat.be/, accessed on 7 July 2022), a global database of technical and natural disasters, 1877 large-scale geohazards occurred worldwide between 1 January 1990 and 7 July 2022. These disasters killed 2.43 million people, left 25.74 million people homeless, and caused $862 million in damages. Japan and China are the countries with the highest losses due to geohazards, which caused approximately $392 million and $114 million in damages, respectively (Figure 1a). As shown, the number of geohazards increased from 1990 to 2000. Asia and the Americas, which account for 55.5% and 22.9% of the total number of geohazards worldwide, respectively, have suffered the most from geohazards (see Figure 1c).

**Figure 2:**
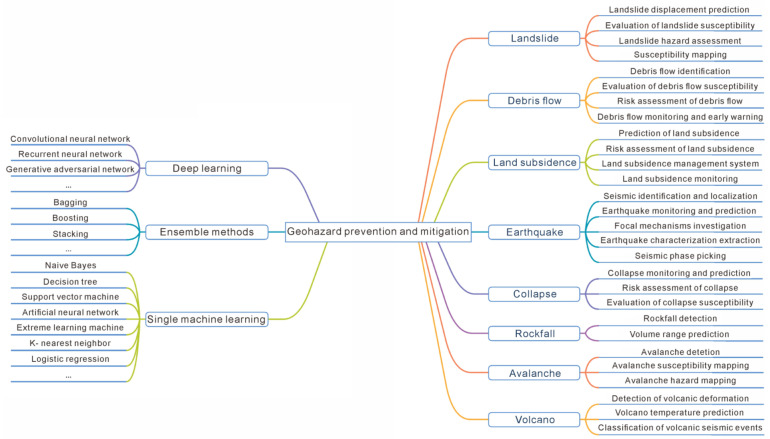
Main AI algorithms and applications in geohazard prevention mitigation.

Great efforts have been made in geohazard prevention and mitigation [2,3,4]. However, geohazards are characterized as complex and uncertain [5,6]; thus, challenges remain for researchers and practitioners [7]. Recently, artificial intelligence (AI) has become popular among researchers and practitioners and has led to considerable advances in geohazard research. Affected by multiple triggering factors [8,9], the monitoring data of the geohazard are usually characterized with complex and nonlinear relationships. For example, due to seasonal rainfall and periodic reservoir fluctuation, the landslide movements in the Three Gorges Reservoir area are characterized with step-like deformation, which makes the displacement predictions remain as challenges. AI is able to analyze these complex and nonlinear characteristics well by establishing a mapping between the input feature data and the output final results [10]. AI has proven its capability in dealing with high-dimensional and large-scale datasets by providing satisfactory predictions [11]. Moreover, AI, a data-driven approach, relies less on expertise and clear understanding of physical processes [12]. Based on previous review works [13,14], AI is widely used in the geohazard field [15,16] (see Figure 2). For example, Kalantar et al. and Xia et al. [17,18] modeled landslide susceptibility using the support vector machine (SVM) algorithm, logistic regression (LR) algorithm, and artificial neural network (ANN) algorithm. Ghorbanzadeh et al. [19] evaluated the application of deep learning (DL) in landslide identification. Zhang et al. [20] used ML algorithms such as decision tree (DT) and random forest (RF) to map landslide susceptibility. Mousavi et al. [21] proposed a DL model for simultaneous seismic detection and phase selection. Wu et al. [22] used AI algorithms such as SVM for tunnel collapse risk assessment. Choubin et al. [23] adopted AI algorithms such as multivariate discriminant analysis for the prediction of avalanche hazards. Valade et al. [24] implemented intelligent monitoring of global volcanic activity using AI techniques on multisensory satellite-based imagery from Sentinel-1. The rapid development of AI research in geohazards has led to a rapid increase in the number of publications on the subject. This makes it difficult for researchers and practitioners to keep abreast of cutting-edge research information and the overall status of research in this field, which can easily lead to meaningless and repetitive studies. To solve this problem, a scientometric analysis and review of the current state of recent research in this area is necessary.

Several researchers have previously conducted review studies in this field. For example, Dikshit et al. [13] provided a qualitative analysis of the application of AI in geohazards highlighting the direction of development in this field. Huang et al. and Merghadi et al. [25,26] analyzed the application of DL in the field of landslide susceptibility evaluation. Xie et al. [27] provided an overview of the applications and prospects of machine learning (ML) in the field of seismic research. Despite their important contributions to the development of the field, these review studies have some limitations. Most of these review studies are qualitative or are limited to the application of a particular AI to a certain type of geohazard; thus, there is a lack of quantitative and comprehensive review studies of the development of AI in geohazard research. In addition, the current review studies in the field do not include an analysis of the publication characteristics of existing papers, the main authors, institutions, and countries or the studies related to the identification of hot research in the field. Therefore, the current review studies do not provide a comprehensive and objective description of the current state of research of AI in the field of geohazards.

A scientometric analysis, which refers to the quantitative study of science and communication in science [28], is promising for addressing the abovementioned limitations as it can handle large volumes of publications; thus offering a visualization of research networks of key scholars, institutions, fundamental articles, and salient topics. Therefore, scientometric reviews have been applied to various research fields [29,30,31,32]. However, so far, no previous reviews have conducted the scientometric analysis of AI-based geohazard research by identification of the salient term and research trend and mapping interconnection.

To fill the gap in quantitative analysis research in geohazard reviews and promote development, quantitative analysis methods are used in this study to analyze and summarize the development of AI in geohazard research from 1990 to 2022. This study contributes to the development of the field of geohazards by objectively presenting the current research status and future directions of AI in this field. The main researchers, institutions, countries, and hot research topics are identified. The advantages and limitations of popular AI algorithms in the field of geohazards are analyzed, and future directions are discussed.

## 2. Materials and Methods

**Figure 3:**
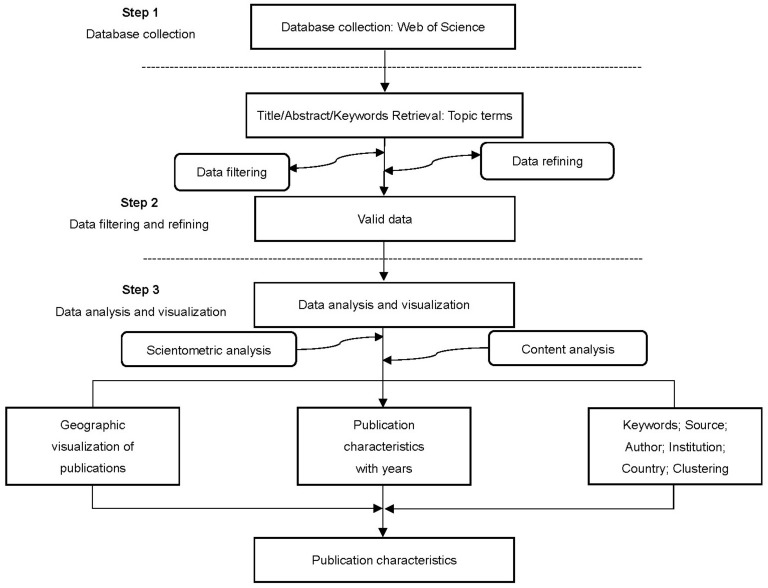
Flow chart for scientometric analysis of AI for geohazard research.

Scientometric analysis is a method of scientific analysis that shows the logic and connections between documents by mapping, mining, ranking, and analyzing them [33]. Various techniques, such as BibExcel, HistCite, and CiteSpace are available to achieve this goal. CiteSpace (version 5.4. R1 64 bit) [34,35] was chosen in this study because the clarity and interpretability of the resulting visualizations are better than those of other scientometric analysis tools. In the present study, a scientometric analysis of AI for geohazard research was performed based on the following three procedures (see Figure 3).

**Data collection:** Web of Science (http://apps.webofknowledge.com, accessed on 7 March 2022) is a comprehensive database with high-quality citation analysis [36] that is based on high-quality citation data, publication standards, and expert judgment. This database is of higher quality, contains more specialized data than other databases (such as Scopus and Google Scholar [37]) and can support a longer period of citation analysis. Thus, in this study, Web of Science was adopted for data collection. A search was performed for the topic (TS) query in Web of Science using the following formula “TS = ((fuzzy sets or naive Bayes or linear regression or random forests or gradient boosting or reinforcement learning or meta heuristics or AI or artificial intelligence or optimization algorithm or machine learning or deep learning or computational intelligence or decision tree or prediction model) AND (geohazard or landslide or slope or rockfall or collapse or earthquake or debris flow or hazard or tsunami))”. Based on the literature search publications in the English language were selected. The year of publication ranged from 1 January 1990 to 1 January 2022, and the subject categories were refined to GeoScience Multidisciplinary and Engineering Geological. A total of 9226 documents were retrieved for scientometric analysis.

**Data filtering and refining:** Subject terms were identified, subject searches were performed in the Web of Science database (“title, abstract, author keywords, and KeyWords Plus”), and Boolean operators (OR/AND) were used to expand the search and exclude irrelevant papers. After filtering and refining the search results to determine the time frame, the search results were downloaded and prepared for the next step of the analysis.

**Data analysis and visualization:** After filtering and refining the papers, the data were visually represented by using the visualization tool CiteSpace. Cluster analysis, an exploratory data mining technique, was adopted for the identification of the salient term and context, research trend, and interconnection. Log-likelihood ratio was used as the clustering index due to advantages of high-quality classification with high intra-class similarity and low inter-class similarity. A cluster overlap indicates that there are relationships between keywords of these different clusters. CiteSpace was used as a tool for performing cluster analysis. A visualization map generated by CiteSpace consists of color-coded nodes and links that describe co-citations or cooccurrences between these nodes. Each representative node, which is made up of a “tree ring” of different colors, denotes one specific item (e.g., country, institution, keyword, author, cited reference, or cited journal). The spectrum of colors denotes the temporal order: oldest in blue and newest in orange. The size of the ring represents the frequency of the corresponding item in a particular year. A red ring present in a particular year denotes a burst, that is, a surge of occurrences or citations in that year. Based on the data visualization, scientometrics and content analysis of the search results in the field are performed and discussed and the results are derived.

## 3. Results

### 3.1. Analysis of Publication Characteristics

#### 3.1.1. Publication Distribution Characteristics

**Figure 4:**
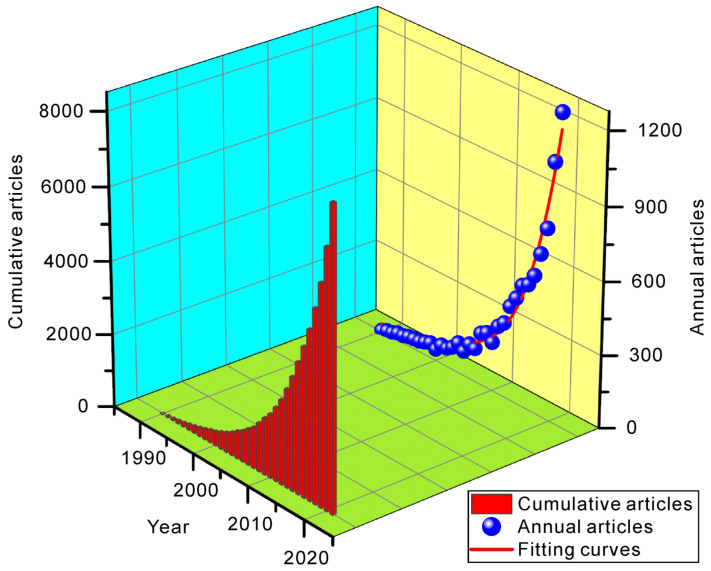
Characteristics of publications of AI for geohazard research by year.

The characteristics of the publications on AI for geohazard research over time are shown in Figure 4. As shown, since 1990, the number of papers published in this field has continued to increase. After entering the 21st century, with the rapid development of AI technology, AI technology in the field of geohazards has developed quickly. As shown in Figure 1b, after 2000 there was an overall decreasing trend of geohazards. At the same time the research of AI in the field of geohazards began to grow rapidly, which to some extent reflects the help of AI in geohazard prevention and mitigation.

The spatial distribution characteristics of publications are shown in Figure 1a. The circles of different colors and sizes in the figure indicate the total number of papers published in the countries where the circles are located. As shown, China and the United States, with a total of 2320 publications and 1993 publications, respectively, are the two countries with the highest total number of publications in this field. In addition, the total number of national papers is positively related to geohazard losses in that country.

#### 3.1.2. Publication Source Characteristics

To provide researchers with references to authoritative publication sources and to facilitate access to relevant and cutting-edge papers, the top 15 sources of AI for geohazard research are shown in Table 1. The sources of the top 15 papers are all journals and there are no conference papers; these data suggest that researchers prefer to publish their papers in journals rather than academic conferences. In addition, the papers from the top 15 sources account for 48.46% of the total number of papers. Among them, **Natural Hazards** and **Remote Sensing**, with 5.96% and 4.58% of the total number of papers, respectively, are among the top two source journals in this field. Among the top 15 sources, **Geomorphology** (17,656 citations), **Journal of Hydrology** (13,417 citations), and **Catena** (12,810 citations) are the most cited. Those results correspond well with the bibliometric review of Wu et al. [38] in the field of AI. Among the top 15 sources, **Geomorphology** (61.52), **Earthquake Spectra** (50.59), and **Catena** (47.62) have the highest average number of citations. **Geomorphology** and **Catena** not only have a high total number of citations but also a high average number of citations. Therefore, they are considered to be the most active journals in this field.

#### 3.1.3. Publication Keyword Characteristics

**Figure 5:**
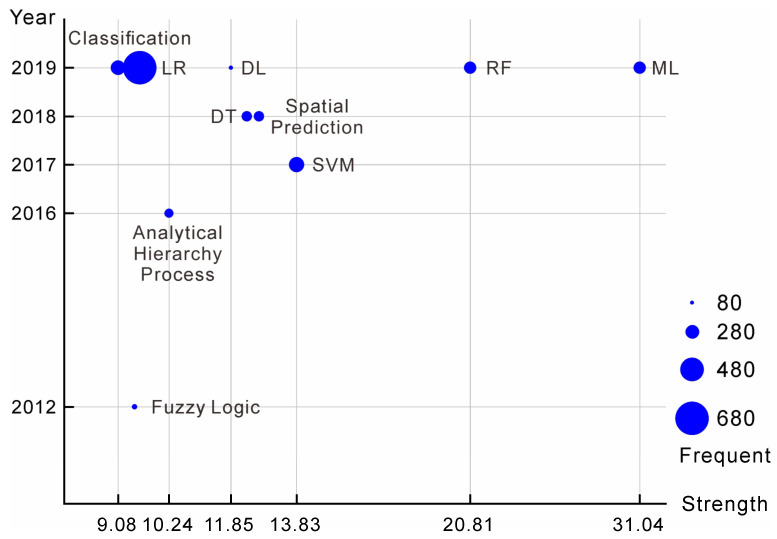
Citation burst of AI in geohazard research during 1990–2022 (logarithmic scale).

Figure 5 shows the 10 keywords with the strongest citation burst of AI research citations in the field of geohazards, representing the main interests of researchers in the field. As shown, researchers have been interested in researching AI techniques in geohazards since 2012 when fuzzy logic was a popular research topic in the field. New research hotspots have gradually emerged. In 2016, the analytical hierarchy process appeared. In 2017, SVM became the third most cited keyword in this citation burst. Subsequently, LR, DL, and many other ML algorithms began to be widely applied in geohazards and became hot topics in the field. Furthermore, the different sizes of circles in Figure 5 indicate different occurrence frequencies; the larger the circle is, the higher the occurrence frequency. Among them, LR appeared 671 times and was the most popular keyword in the field. Moreover, an increasing number of AI algorithms have been applied to the field of geohazards by researchers.

### 3.2. Analysis of Authors, Institutions, and Countries

#### 3.2.1. Most Productive Authors in AI Research in the Field of Geohazards

The affiliations of the top 15 most productive authors in terms of AI research in the field of geohazards and their H-index, total number of papers, and total number of citations are shown in Table 2. As shown in this table, each of these authors published 26 or more papers. These authors have published a cumulative total of 732 papers, which accounts for 7.93% of the papers published by researchers worldwide. Six of these researchers are affiliated with Asian institutions, with three researchers from China and three from Iran. Therefore, AI in the field of geohazards is considered to be developing rapidly in Asia, with China and Iran being the main participating countries in this field of research.

The most productive authors are Pradhan, Biswajeet (136); Dieu Tien Bui (70); and Pourghasemi, Hamid Reza (65); who are also the most cited authors. Six of the top 15 authors have a higher number of average citations (i.e., higher than 69.30) than the rest. They are Pourghasemi, Hamid Reza (97.42); Pradhan, Biswajeet (96.66); Shahabi, Himan (84.60); Dieu Tien Bui (80.06); Rahmati, Omid (76.93); and Hong, Haoyuan (69.94). Four authors have an H-index above 60: Pradhan, Biswajeet (94); Dieu Tien Bui (68); Pourghasemi, Hamid Reza (66); and Lee, Saro (64). Six authors have a relative citation impact greater than 1 relative to the other top 15 authors. They are Pourghasemi, Hamid Reza (1.41); Pradhan, Biswajeet (1.39); Shahabi, Himan (1.22); Dieu Tien Bui (1.16); Rahmati, Omid (1.11); and Hong, Haoyuan (1.01). According to these data, Pradhan, Biswajeet; Dieu Tien Bui; and Pourghasemi, Hamid Reza have better performances under all parameters. Therefore, they are considered to be strong influential researchers in the field.

#### 3.2.2. Most Productive Institutions in Terms of AI Research in the Field of Geohazards

Among 9226 scientometric records of AI studies in the field of geohazards, 841 institutions are identified. Table 3 shows the data related to the top 15 most productive institutions. At each of these institutions, 83 or more papers have been published. These papers (1806 total) account for 19.58% of the cumulative number of papers in the field. The institutions with the most published papers are the Chinese Academy of Sciences (384), China University of Geosciences (158), and U.S. Geological Survey (156). The Chinese Academy of Sciences has not only published the most papers but also has the highest total citations and is one of the most active research institutions in AI research in the field of geohazards. Those results correspond well with the bibliometric review of Ho and Wang [39] in the field of AI.

The average citations per paper for all papers related to AI research in the field of geohazards from the top 15 institutions is 28.46%. Six institutions have higher average citations per paper than the others. These institutions include the U.S. Geological Survey (57.23); University of California, Berkeley (50.91); Sejong University (42.82); Duy Tan University (36.02); Islamic Azad University (35.74); and China Earthquake Administration (28.60). From 1990 to 2022, the relative citation impact of the top 15 most productive institutions relative to the total global research output of AI in geohazards was 1.00. Five institutions exceeded this relative citation impact, including the U.S. Geological Survey (2.00); University of California, Berkeley (1.78); Sejong University (1.5); Duy Tan University (1.26); and Islamic Azad University (1.25).

According to average citations per paper and relative citation impact, the U.S. Geological Survey; University of California, Berkeley; and Sejong University are considered to be the most active institutions in this field.

#### 3.2.3. Top Countries in Terms of AI Research in the Field of Geohazards

Information on publications by country and region is closely related to publication characteristics but reflects different information (see Table 4). This table shows that China is the country with the most publications in this field during 1990–2022, with 2349 historical publications. The United States and Italy rank second and third with a total of 1993 and 894 publications, respectively. In addition, the United States has the highest total citations with 61,656 historical citations, followed by China (41,179) and Italy (27,388). Malaysia has the highest average citations per paper with an average of 75.71, followed by Norway and Vietnam with an average of 56.19 and 41.37 citations per paper, respectively. In the area of intercountry cooperation, the United States is the most influential country in this field and has the highest number of collaborations among 10 countries, including China, Italy, and the United Kingdom. In addition, the United States and China are the closest collaborators, with 279 collaborations.

**Figure 6:**
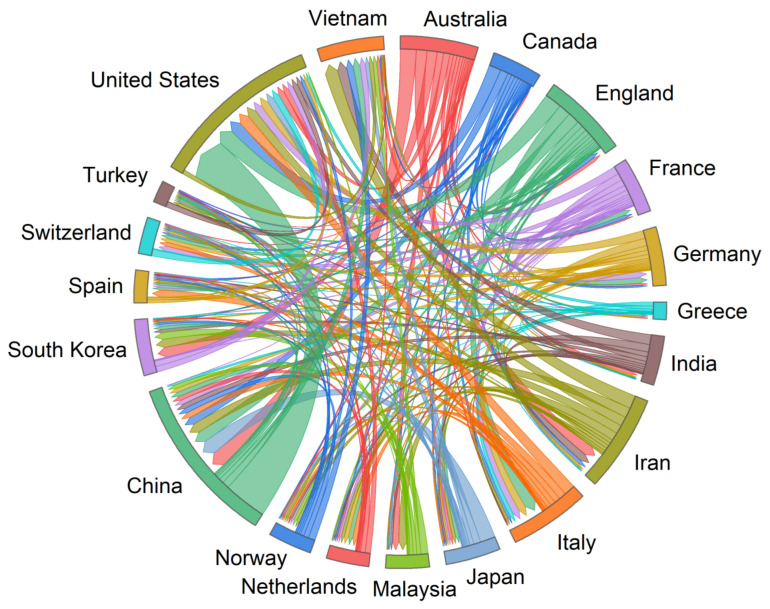
Cooperation between countries and regions.

Figure 6 shows the cooperation of major countries and regions in this field. The figure clearly shows that China, the United States, Italy, and Iran are the countries with the most AI studies in geohazard research. They are also the countries with the highest number of papers published. Among them, China has the highest number of publications and the second highest number of collaborations with other countries after the United States. The United States is the second most published country and has the most collaborations with other countries. In addition, China and the United States are the countries that cooperate most closely with each other. Italy has published more papers than Iran but has collaborated less with other countries than Iran. These results indicate that China and the United States are the two most representative countries in AI research in the field geohazards. Those results correspond well with the previous bibliometric review of Ho and Wang and Wu et al. [38,39]. Additionally, Italy and Iran also have a productive role in the AI-based geohazard research.

### 3.3. Identification of Salient Research Clusters

**Figure 7:**
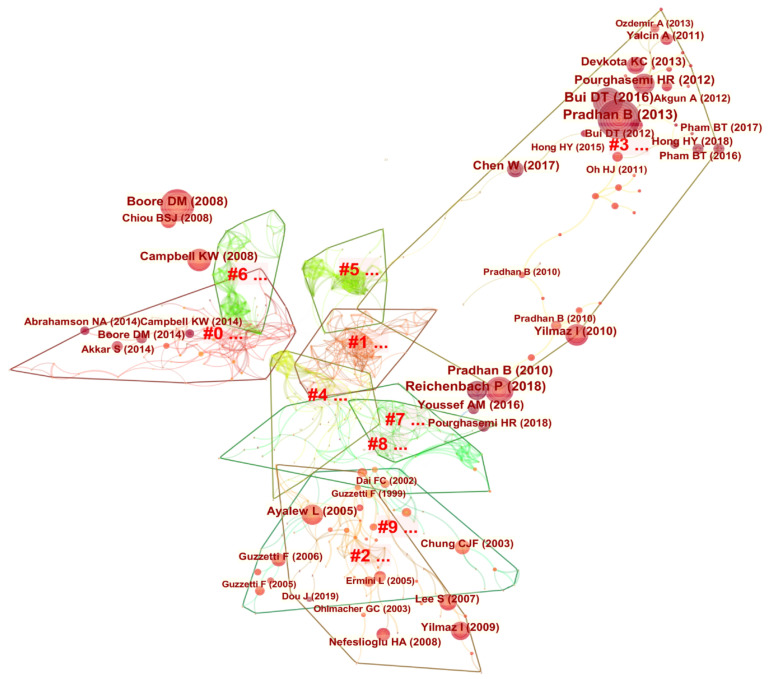
Research cluster network for AI in geohazard research.

In this study, we selected papers ranked in the top 40% of references each year as the prominent research clusters for identifying the development of AI in geohazard research. By applying the log-likelihood ratio algorithm, 10 prominent research clusters were identified based on the keywords of the top-cited references (see Table 5). The identified clusters for #0 to #9 are ground motion, DL, GIS, landslide, impact, segment linkage, prediction, root reinforcement, debris flow, validation, respectively. Figure 7 and Table 5 show the prominent research clusters obtained based on the Web of Science search results. The silhouette value, a measure of the homogeneity of individual clusters, ranges from −1 to 1. The clustering results are considered convincing only when the silhouette value is greater than 0.5. As shown in Table 5, the silhouette values determined in this study ranged from 0.81 to 1, which indicates that the clustering results are convincing and that the members of each cluster have good consistency. For brevity, only the first five clusters (#0 to #4) were analyzed in this study and are discussed below.

**Ground motion:** The largest cluster (#0) is labeled ground motion, with a total of 70 members and a silhouette value of 0.81. A representative paper is that by Boore and Atkinson [40]. Ground motion usually refers to the surface movement of an area caused by an earthquake or explosion that results from waves generated by the sudden sliding of a fault or the sudden appearance of pressure from an explosion source and propagates along the surface of the Earth.

**Deep learning:** The second largest cluster (#1) is labeled DL, with 64 members and a silhouette value of 0.91. A representative paper is that by Bui et al. [41]. DL, a major branch of ML, is an algorithm for learning representations of information based on ANNs [45]. With the development of DL, its powerful nonlinear data processing capability has received increasing attention from geohazard researchers. DL is very powerful in geohazard processing and is effective in information extraction. It has since been introduced into geohazard analysis and prevention [14], including landslide and mudflow detection, seismic data interpolation and noise reduction.

**GIS:** The third largest cluster (#2) is labeled GIS, with 61 members and a silhouette value of 0.97. A representative paper is that by Guzzetti et al. [42]. GIS, or geographic information systems, is a comprehensive discipline of geography, cartography, and computer technology and is now widely used in the field of geohazards. GIS technology has contributed to quantitative studies of geohazard risk assessment and mapping. It has made important contributions in delineating geohazard susceptibility and sensitivity maps, land planning and utilization, and disaster loss reduction [46].

**Landslide:** The fourth largest cluster (#3) is labeled landslide, with a total of 54 members and a silhouette value of 0.91. Landslides are one of the most common geohazards, causing large economic losses and safety threats to people every year. Representative papers include those by Pradhan [43], Tien Bui et al. [47], and Pourghasemi et al. [48]. These papers consider specific applications of AI in landslide hazards. AI is commonly used in landslide hazards such as displacement prediction and susceptibility mapping.

**Impact:** The fifth largest cluster (#4) is labeled impact, with 47 members and a profile value of 0.92. The impact label includes both the impact factors and the impacts caused by geohazards. In representative papers by Kim et al. and Ma et al. [44,49], the impact factors of geohazards were studied. Claessens et al. [50] studied the impact of geohazards.

### 3.4. Top Algorithms and Future Trends in AI Research of Geohazards

In addition to the keyword characteristic analysis of publications, the 10 keywords with the strongest keyword citation burst are all related to AI technology. Seven of these keywords are AI algorithms. This also indicates that AI algorithms have become an important method in geohazard research. Therefore, it is necessary to summarize and analyze the AI algorithms commonly used in geohazard research. Table 6 shows a brief summary and some advantages and limitations of some common AI algorithms in the field of geohazards. Among them, NB, DT, and SVM are the classic ML algorithms. These common single ML algorithms had seen citation outbreaks one after another in 2017 and are widely used by researchers of geohazards. Recently, DL methods, including autoencoders and convolutional and recurrent neural networks, have been widely used by researchers because of their greater processing power of raw natural data [51] and higher accuracy of qualitative hazard prediction [16] than traditional ML methods. DL is the second largest cluster in the cluster analysis and a silhouette of 0.91 is a convincing result. Therefore, it can be considered that DL is one of the trends of AI in geohazards.

## 4. Future Directions

AI has been extensively applied to geohazard research, yielding tremendous success. Based on the scientometric analysis of the literature to date, we recommend the following aspects should be addressed for AI-based geohazard research.

### 4.1. Establishment of Benchmark Database

AI modeling is driven by data [103]; therefore, the quantity and quality of the data may directly affect the performance of AI [14]. However, some fundamental constraints remain for data acquisition and preparation. Firstly, the high cost of monitoring equipment limits the coverage of field monitoring and limits researchers’ access to high-quality field data. Another impediment is the lack of large and generalized geohazard datasets. Although tens of thousands of papers have been published for AI-based geohazard research, it is difficult to extract and utilize openly available, curated, and labeled training data. Generally, researchers from different institutions often use different datasets and research methods for their studies, with the terminology and data completeness in the papers varying tremendously. This has led to strong calls from researchers for the establishment of a benchmark database, data sharing, and standardization of data reporting [104] which will be an important boost for the development of AI-based geohazard researches. Some researchers have already started data sharing work. For example, Ji et al. [105] shared a large landslide dataset (http://study.rsgis.whu.edu.cn/pages/download/, accessed on 4 October 2022) containing landslide images, landslide boundary information, landslide area DEM data, etc. Mousavi et al. [100] contributed a large number of high-quality seismic analysis datasets (https://github.com/smousavi05/STEAD, accessed on 4 October 2022) which contain local seismic waveforms, seismic noise waveforms, and no seismic signals. These publicly available high-quality datasets can be used as benchmark datasets for the evaluation of the performance of different AI algorithms in this field and provide a reproducible evaluation environment. In addition, a standardized baseline database not only provides researchers with high-quality datasets but also eases the work of researchers in data management [106]. Therefore, a standardized geohazard benchmark database is desired by researchers.

### 4.2. Integration of AI with Physical Processes

AI techniques provide good performances in geohazards such as landslide susceptibility evaluation [20], earthquake identification and phase selection [21], and volcanic activity monitoring [24]. However, researchers and practitioners still face challenges in enhancing the reliability [107]. To improve the reliability of AI, some researchers have attempted to integrate AI and physical processes to embody the powerful data processing capabilities of AI techniques and the reliability of physical processes in an ensemble algorithm [108]. For example, Jiang et al. [108] proposed an algorithm to improve the geoscientific knowledge of AI. Depina et al. [109] used an algorithm for the study of unsaturated groundwater flow using a combination of AI and physical processes. The reliability has been enhanced by adopting data-driven components to improve the unrepresentable parts of physical processes and integration of the evolution of physical processes in AI algorithms.

### 4.3. Auto ML

A strong mastery of expert knowledge is required for AI-based geohazard research. A general workflow for AI modeling usually consists of data preprocessing, feature engineering, selection of a machine learning model, and optimization of the associated hyperparameters [110,111]. Reducing the requirement of expert knowledge and automating all the processing steps is a common expectation among researchers. Some researchers have offered auto ML platforms that have somewhat overcome the problems of algorithm selection and hyperparameter optimization, reducing the need for expertise in AI algorithms. For example, Auto-sklearn optimized hyperparameter selection using a Bayesian algorithm and automated policy selection using meta-learning and integration structures [112]. Auto-WEKA implements the automatic selection of algorithms and hyperparameter optimization based on Bayesian optimization techniques [113]. These auto ML platforms have proven their capacity in the fields of medicine [114], mechanics [115], and geoscience [116].

### 4.4. Uncertainty Quantification

AI analyzes geohazard data by building corresponding models to predict the occurrence of geohazards and provide evidence and suggestions for its prevention and mitigation. In this process, the uncertainties existing in the data and models may bias the analysis results. Data uncertainty is generated due to class overlap and noise in the training data and is non-approachable due to limitations in how the data are collected. Epistemic uncertainty results from errors caused by model inference or model performance [117]. With the widespread use of AI in geohazards, it is becoming more and more crucial to evaluate the validity and reliability of AI systems before using their analysis results.

Currently, accurate uncertainty quantification is the key to enhance the reliability and accuracy of AI analysis results and the future direction of AI in the field of geohazards. A few researchers have started research on uncertainty quantification. The most common approaches can be divided into Bayesian uncertainty quantification that focuses on specifying the training set to approximate the posterior probability distribution, such as Monte Carlo [118] and Markov Chain Monte Carlo [119], and ensemble uncertainty quantification that obtains improved accuracy by combining multiple models [120] such as deep ensemble [121] and Dirichlet Deep Networks [122].

### 4.5. Interpretable AI

Some AI algorithms cannot provide a reasonable interpretation for their results which makes researchers and practitioners distrust results obtained from AI. This has limited the development of AI-based geohazard research to a large extent and has brought increasing attention to interpretable AI [123]. Based on previous studies, research methods for interpreting AI techniques are maturing [124,125], terminology and metrics are being harmonized [126,127], and there is some development in the evaluation of interpretable AI and interpretation of AI. Some primary research methods are currently being used to study AI “black boxes”; for example, by decomposing model components into small parts that we can explain [128] and by visualizing the weights of different models to improve the interpretability of DL for seismic monitoring and phase selection [21]. Future works should include overcoming the obstacles to development caused by the uncertainty of quantitative AI interpretation methods, causal interpretation, feature dependence, and other problems [129].

## 5. Conclusions

AI has been extensively applied to geohazard research and yielding tremendous success. The present study performed a scientometric-assisted review for AI-based geohazard research by visualization of the research status quo and identification of the salient term and context, research trend, and mapping interconnection based on 9226 scientometric records. The analysis of the research publication trend indicates that AI has obtained continuous development in geohazard research over the past 30 years and entered a period of rapid growth beginning in 2000. An analysis of publication source characteristics has revealed that **Natural Hazards** and **Remote Sensing** are the top two source journals. **Geomorphology** and **Catena** are considered to be the most active journals in this field. The analysis of keyword features revealed that ML is a popular research method in this field. **Pradhan, Biswajeet**; **Dieu Tien Bui**, and **Pourghasemi, Hamid Reza** are among the three most productive researchers in this field. Three organizations including the **U.S. Geological Survey**; **University of California, Berkeley**; and **Sejong University** are considered to be the most productive institutions in this field. China, the United States, and Italy are the countries with the highest number of publications and the highest number of total citations among all countries. Identification of salient research clusters indicates that ground motion, **DL**, **GIS**, and landslides are current research hotspots.

Future studies on AI-based geohazard research themes may focus on the establishment of benchmark database, integration of AI with physical processes, Auto ML, uncertainty quantification and interpretable AI.

This scientometric review offers useful reference points for early-stage researchers and provides valuable in-depth information to experienced researchers and practitioners in the field of geohazard research. This scientometric analysis and visualization are promising for comprehensively reflecting the global picture of AI-based geohazard research and are potential for visualization the emerging trends in other research fields.

## Figures and Tables

**Table 1:** Top 15 source journals according to the number of publications in AI for geohazard research (1990–2022).

No.	Source	Total Papers	Total Citations	Average Citations per Paper	Percentage of Total Papers
1	Natural Hazards (https://www.springer.com/journal/11069, accessed on 3 September 2022)	550	12,286	22.34	5.96%
2	Remote Sensing (https://www.mdpi.com/journal/remotesensing, accessed on 3 September 2022)	423	6376	15.07	4.58%
3	Journal of Hydrology (https://journals.elsevier.com/journal-of-hydrology, accessed on 3 September 2022)	314	13,417	42.73	3.40%
4	Bulletin of Earthquake Engineering (https://www.springer.com/journal/10518/, accessed on 3 September 2022)	306	6068	19.83	3.32%
5	Soil Dynamics and Earthquake Engineering (https://www.sciencedirect.com/journal/soil-dynamics-and-earthquake-engineering, accessed on 3 September 2022)	306	4060	13.27	3.32%
6	Environmental Earth Sciences (https://www.springer.com/journal/12665, accessed on 3 September 2022)	298	7798	26.17	3.23%
7	Engineering Geology (https://www.sciencedirect.com/journal/engineering-geology, accessed on 3 September 2022)	290	11,417	39.37	3.14%
8	Geomorphology (www.elsevie r.com/locate/geomorph, accessed on 3 September 2022)	287	17,656	61.52	3.11%
9	Catena (www.elsevier.com/locate/catena, accessed on 3 September 2022)	269	12,810	47.62	2.92%
10	Natural Hazards and Earth System Sciences (https://www.natural-hazards-and-earth-system-sciences.net/, accessed on 3 September 2022)	264	6611	25.04	2.86%
11	Landslides (https://www.springer.com/journal/10346, accessed on 3 September 2022)	262	8970	34.24	2.84%
12	Arabian Journal of Geosciences (https://www.springer.com/journal/12517, accessed on 3 September 2022)	243	3298	13.57	2.63%
13	Earthquake Engineering & Structural Dynamics (https://onlinelibrary.wiley.com/journal/10969845, accessed on 3 September 2022)	233	7756	33.29	2.53%
14	Earthquake Spectra (https://journals.sagepub.com/home/eqs, accessed on 3 September 2022)	213	10,776	50.59	2.31%
15	Geophysical Research Letters (https://agupubs.onlinelibrary.wiley.com/journal/19448007, accessed on 3 September 2022)	213	5269	24.74	2.31%

**Table 2:** Top 15 most productive authors for AI research in the field of geohazards (1990–2022).

No.	Name	Institution, Country	Total Papers	Total Citations	Average Citations per Paper	H-Index	Related Citations Impact
1	Pradhan, Biswajeet	University of Technology Sydney, Australia	136	13,146	96.66	94	1.39
2	Dieu Tien Bui	University of South-Eastern Norway, Norway	70	5604	80.06	68	1.16
3	Pourghasemi, Hamid Reza	Shiraz University, Iran	65	6332	97.42	66	1.41
4	Chen, Wei	Xi’an University of Science and Technology, China	63	4104	65.14	53	0.94
5	Lee, Saro	Korea Institute of Geoscience and Mineral Resources, KIGAM, Korea	63	3880	61.59	64	0.89
6	Hong, Haoyuan	Universität Vienna, Austria	52	3637	69.94	45	1.01
7	Binh Thai Pham	University of Transport Technology, Vietnam	45	2851	63.36	26	0.91
8	Arabameri, Alireza	Tarbiat Modares University, Iran	32	684	21.38	29	0.31
9	Bradley, Brendon A.	University of Canterbury, New Zealand	32	583	18.22	36	0.26
10	Xu, Chong	Institute of Geology, China Earthquake Administration, China	31	1693	54.61	29	0.79
11	Shahabi, Himan	University of Kurdistan, Iran	30	2538	84.60	52	1.22
12	Xu, Qiang	Chengdu University of Technology, China	30	659	21.97	43	0.32
13	Rahmati, Omid	Agricultural Research, Education and Extension Organization (AREEO), Vietnam	29	2231	76.93	36	1.11
14	Prakash, Indra	Bhaskaracharya Institute for Space Applications and Geoinformatics, India	28	1551	55.39	37	0.80
15	Blaschke, Thomas	Universität Salzburg, Austria	26	1235	47.50	50	0.69

Note: The H-index in the table header means that the author has H papers cited H times.

**Table 3:** Top 15 most productive institutions in terms of AI research in the field of geohazards (1990–2022).

No.	Institution	Total Papers	Total Citations	Average Citations per Paper	Relate Citations Impact
1	Chinese Academy of Sciences (https://english.cas.cn, accessed on 3 September 2022)	384	9088	23.67	0.83
2	China University of Geosciences (https://en.cug.edu.cn, accessed on 3 September 2022)	158	3303	20.91	0.73
3	U.S. Geological Survey (https://www.usgs.gov, accessed on 3 September 2022)	156	8928	57.23	2.00
4	University of Chinese Academy of Sciences (https://english.ucas.ac.cn, accessed on 3 September 2022)	115	1264	10.99	0.38
5	Chengdu University of Technology (http://www.cdut.edu.cn. accessed on 3 September 2022)	101	1549	15.34	0.54
6	Tongji University (https://en.tongji.edu.cn, accessed on 3 September 2022)	100	1749	17.49	0.61
7	University of California, Berkeley (https://www.berkeley.edu, accessed on 3 September 2022)	97	4938	50.91	1.78
8	University of Technology Sydney (https://www.uts.edu.au, accessed on 3 September 2022)	93	2583	27.77	0.97
9	Duy Tan University (https://duytan.edu.vn, accessed on 3 September 2022)	91	3278	36.02	1.26
10	University of Tehran (https://ut.ac.ir/en. accessed on 3 September 2022)	88	2027	23.03	0.81
11	Islamic Azad University (https://iau.ae, accessed on 3 September 2022)	86	3074	35.74	1.25
12	China Earthquake Administration (https://www.cea.gov.cn, accessed on 3 September 2022)	85	2431	28.60	1.00
13	Sejong University (https://en.sejong.ac.kr/eng/index.do, accessed on 3 September 2022)	85	3640	42.82	1.50
14	Tarbiat Modares University (https://en.modares.ac.ir, accessed on 3 September 2022)	84	2309	27.49	0.96
15	Kyoto University (https://www.kyoto-u.ac.jp/en, accessed on 3 September 2022)	83	1238	14.92	0.52

**Table 4:** Top 20 countries or regions in terms of number of publications.

Country	Total Papers	Total Citations	Average Citations per Paper	Closest Collaborating Country	Number of Total Collaborators
China	2349	41,179	17.53	United States	279
United States	1993	61,656	30.94	China	279
Italy	894	27,388	30.64	United States	72
Iran	629	19,302	30.69	Vietnam	85
England	572	18,371	32.12	United States	111
Japan	505	11,036	21.85	China	83
India	504	9430	18.71	Vietnam	63
Australia	443	11,376	25.68	China	100
Germany	410	11,086	27.04	United States	51
Canada	409	14,586	35.66	United States	78
France	386	10,326	26.75	United States	51
South Korea	323	10,793	33.41	Australia	65
Turkey	289	9311	32.22	United States	34
Spain	277	7582	27.37	Italy	47
Switzerland	240	7625	31.77	United States	42
Netherlands	239	9496	39.73	United States	35
Vietnam	182	7529	41.37	Iran	85
Greece	174	4913	28.24	Italy	26
Malaysia	171	12,946	75.71	Iran	52
Norway	162	9103	56.19	Vietnam	45

**Table 5:** Research clusters of AI in geohazard research between 1990 and 2022.

Cluster ID	Size	Silhouette	Cluster Label	Representative Document
#0	70	0.81	Ground motion	Boore & Atkinson [40]
#1	64	0.91	Deep learning	Bui et al. [41]
#2	61	0.97	GIS	Guzzetti et al. [42]
#3	54	0.91	Landslide	Pradhan [43]
#4	47	0.92	Impact	Kim et al. [44]

**Table 6:** Summary of popular AI algorithms in the field of geohazards.

**1**	**Naive Bayes**	
	**Summary**	NB classifiers are simple probabilistic classifiers based on the Bayes theorem and the strong (naive) independence assumption between features [52].
	**Advantages**	NB is simple, efficient, and reliable [53]; The NB classifier does not require a complex iterative parameter estimation scheme and is easy to construct [54]; The NB classifier handles correlated noise and irrelevant attributes and has very good robustness [55].
	**Limitations**	NB determines the posterior probability by using the prior probability and data to determine the classification, so there is a certain error rate in the classification decision [56]; NB classifiers require strong independence between attributes [57].
**2**	**Decision Tree**	
	**Summary**	The DT is a basic classification and regression method. The DT model has a tree-like structure and represents the process of classifying instances based on features in a classification problem [58].
	**Advantages**	DT can solve complex problems [59]; It provides expressive representation for learning discrete functions; The time complexity of the decision tree is small [60].
	**Limitations**	A large amount of storage is required to store all classifier results [61]; It is difficult to understand the reasoning process when multiple classifiers are involved in the decision [58]; It is easy to overfit the data [62].
**3**	**Support Vector Machine**	
	**Summary**	SVM is a supervised learning machine proposed by Vapnik et al. [63,64]. It is a powerful tool for solving pattern classification problems and regression problems [65] and has been used in various fields [66,67,68].
	**Advantages**	SVM can achieve better results with fewer samples [25]; SVM is insensitive to dimensionality and outliers [69]; SVM is very robust and accurate [70].
	**Limitations**	The SVM algorithm has high time complexity and memory training complexity [64]; The SVM principle is complex and computationally expensive [71].
**4**	**Artificial Neural Networks**	
	**Summary**	ANNs are algorithmic models inspired by biological neural networks. They are massively parallel systems with a large number of interconnected simple processors [72].
	**Advantages**	ANNs have significant advantages in data classification and regression [73]; ANNs are not constrained by predefined mathematical relationships between variables [74]; ANNs have a powerful ability to handle complex nonlinear problems.
	**Limitations**	The model is considered a black box, making it difficult to understand the internal mechanism [74]; ANNs have a high demand for computing resources; ANNs can easily fall into the local minima and sometimes it is difficult to adjust the structure [75].
**5**	**Extreme Learning Machine**	
	**Summary**	The ELM is a single-layer feedforward neural network that overcomes the difficulty of parameter initialization. It is one of the most widely used algorithms for predicting time series data [76].
	**Advantages**	The theoretical basis of an ELM is relatively simple [76]; ELM can achieve global minimum optimization and has powerful generalization [77]; ELM computes much faster than other feedforward neural networks.
	**Limitations**	An ELM with a fixed number of hidden layer nodes reduces model prediction accuracy [78]; When the training dataset is relatively small ELM has more erroneous results [76].
**6**	**K-Nearest Neighbor**	
	**Summary**	KNN is a nonparametric method that is considered one of the top 10 data mining algorithms because of its simplicity, efficiency, and implementation power for classification [79].
	**Advantages**	The structure of the KNN algorithm is relatively simple and has good portability [80]; KNN algorithms are powerful in classification with good effectiveness and implementation [79].
	**Limitations**	Outliers in KNN algorithms can have a large adverse effect on the results [79]; The problems of similarity measurement of two data points and K-value selection in the KNN algorithm still need to be solved [81].
**7**	**Logistics Regression**	
	**Summary**	LR analysis is a statistical technique for analyzing the relationship between an independent variable and two dependent variables (dichotomous variables) and is widely used in various fields [82,83].
	**Advantages**	A regression relationship is formed between the dependent variable and one or more independent variables [84]; LR algorithms are independent of the data distribution [85]; Continuous explanatory variables can be used [86].
	**Limitations**	LR is more sensitive to multiple linear data [87]; A large sample size is required for goodness-of-fit measurements [59].
**8**	**Ensemble Methods**	
	**Summary**	EMs refer to the combination of individual AI models into one model that has higher accuracy and stronger generalization ability than the individual AI models [88,89].
	**Advantages**	The results of the integrated approach are more accurate than those predicted by individual models [90,91,92]; EMs can avoid overfitting and local optima [93]; EMs have better generalization ability than a single AI algorithm [88,94].
	**Limitations**	EMs tend to ignore local clustering diversity [95]; The accuracy of EMs is determined by the choice of the base model [88]; Much maintenance is required.
**9**	**Deep Learning**	
	**Summary**	DL is a branch of machine learning based on ANN [96]. It has excellent performance in processing a large amount of high level data and has a wide range of applications in various fields [97,98,99].
	**Advantages**	DL has powerful feature learning and expression capabilities [100]; DL is highly efficient in processing high-dimensional data [96]; DL has more frameworks to use and is more compatible.
	**Limitations**	DL requires much data and computing power and the computational cost is high [101,102]; DL requires high hardware requirements due to its high computing power.

## Data Availability

Not applicable.

## Abbreviations

AI: artificial intelligence; ANN: artificial neural network; DL: deep learning; DT: decision tree; EM-DAT: Emergency Events Database; EM: ensemble method; ELM: extreme learning machine; KNN: k-nearest neighbor; LR: logistic regression; ML: machine learning; NB: naive Bayes; RF: random forest; SVM: support vector machine.
